# Programmable nano-optics and photonics

**DOI:** 10.1515/nanoph-2024-0252

**Published:** 2024-05-14

**Authors:** Tian Gu, Arka Majumdar, Jinghua Teng

**Affiliations:** Materials Research Laboratory, 2167 Massachusetts Institute of Technology, Cambridge, MA, USA; Department of Electrical and Computer Engineering, 7284 University of Washington, Seattle, WA, USA; Institute of Materials Research and Engineering, Agency for Science, Technology and Research, Singapore, Singapore

This special issue of *Nanophotonics*, titled “Programmable Nano-optics and Photonics,” highlights cutting-edge advancements in the field that represents a paradigm shift from traditional optical systems with fixed-functionality to dynamically tunable systems. This transformation is marked by enhanced performance and the ability to actively manipulate light propagation, processing, and interaction with matter in unprecedented ways. This issue gathers contributions from leading experts worldwide, featuring groundbreaking research and perspectives that span from theoretical frameworks to advanced applications, illustrating the potential of programmable nano-optics and photonics on both free-space and integrated photonics platforms. In this Editorial, we spotlight the key contributions that illustrate the breadth and depth of this rapidly evolving field.

A comprehensive review on recent progress towards large-scale programmable silicon photonics for signal processing is provided by Xie et al. [1]. These high-performance photonic integrated circuits are crucial for applications including microwave photonics, optical communications, computing, and quantum photonics, reflecting their critical role in future optical systems. Sreekanth et al. furnish an in-depth review on the development of tunable Fano resonant optical coatings using phase change materials. The article further highlights their applications in dynamic structural coloring as well as spectrum splitting for improving photovoltaic efficiency of solar cells [2].

Ullah et al. discuss chalcogenide-based nonvolatile photonic memories using layered polymorphs, which offers an alternative to conventional memory technologies for applications in optical computing [3]. Chen et al. outline how chalcogenide phase-change materials can advance programmable terahertz metamaterials, focusing on their integration into 6G communication technologies [4].

Theoretical limits of multifunctional nanophotonic responses are explored by Shim et al. The study establishes a general framework for optimizing device performance in various design scenarios [5]. Lee et al. discuss advancements in spatio-spectral control within coherent nanophotonics, and demonstrate a reconfigurable and multi-spectral modulator operating within a single element [6]. A method for aligning liquid crystals using nanoantennas for tunable high-performance metasurfaces is presented by Veetil et al., promising enhanced functionality in diverse applications including virtual reality [7].

Xiao et al. introduce a programmable topological metasurface capable of real-time modulation of spatial and surface waves. The demonstration shows versatile wave manipulation capabilities of such a metasurface for potential utilizations in smart sensing and wireless communications [8]. Wang et al. propose a programmable flip-metasurface that controls reflection dynamically while maintaining undistorted transmission over a broad spectral band, opening new avenues in communications and stealth technology [9]. High-resolution non-line-of-sight imaging based on liquid crystal planar optical elements is showcased by Zhao et al., with wide scanning area and high-quality image reconstruction. Such an approach could transform security monitoring and autonomous driving [10]. Yang et al. present a new concept of ultrafast Q-boosting in semiconductor metasurfaces. The proposed approach could provide complete dynamic control over the resonance bandwidth and boost applications in frequency conversion and light trapping [11].

The inverse design of compact, reconfigurable silicon photonic devices using phase-change materials is demonstrated by Wei et al., aiming to improve the performance and tolerance to fabrication errors of photonic devices [12]. Feng et al. introduce scalable and efficient deep learning through integrated multi-operand optical neurons and demonstrate their implementation in image recognition tasks [13]. Zhang et al. present advances in surface plasmon-cavity hybrid states at THz frequencies and active modulation using graphene patterns. This development offers new possibilities for advanced optoelectronic devices for sensing molecules and chemicals, and THz quantum devices, etc. [14].

Hu et al. propose and demonstrate a language-controllable programmable metasurface leveraging large language models. Such an approach can potentially redefine autonomous electromagnetic manipulation [15]. Using elemental antimony, Aggarwal et al. present an integrated microwave photonic processing unit for all-optical RF filtering, which could find wide applications in telecommunications and signal processing [16].

A reconfigurable application-specific photonic integrated circuit for solving partial differential equations is introduced by Ye et al. The work showcases the potential of this approach in addressing complex scientific challenges [17]. Sharma et al. develop a new method to compensate for the crosstalk in optical phased arrays for 2D beam-steering, with programmable amplitude and phase control for each grating antenna element, which can potentially improve applications requiring precise optical control [18].

Almutlaq et al. present closed-loop electron-beam-induced spectroscopy and nanofabrication around individual quantum emitters, which enables high-resolution imaging and enhancement of control over emitter-cavity interactions [19]. Using femtosecond laser writing, Pentangelo et al. present universal photonic processors with high-fidelity and polarization-insensitivity, which will bring various benefits to applications in quantum information processing [20]. Doshi et al. demonstrate high-resolution, direct electron beam patterning of electro-optically active PEDOT:PSS. This work paves the path for novel miniaturized tunable optoelectronic devices [21].

This special issue not only highlights the state-of-the-art advancements in programmable nano-optics and photonics but also sparks discussions on the existing challenges and potential future directions. By compiling innovative research findings and opinions, the issue serves as a vital resource for both seasoned researchers and those new to the field, highlighting the profound potential of programmable optics and photonics to reshape our technological landscape across multiple domains.

The guest editors would like to express their gratitude to all the authors for their outstanding contributions to this special issue. We also extend our sincere thanks to the reviewers, whose high-quality and timely feedback has significantly improved the collection of articles. Special appreciation goes to Dennis Couwenberg, Publishing Editor, and Tara Dorrian, Managing Editor of *Nanophotonics*, for their invaluable support and steadfast commitment throughout the preparation process.
